# Progress towards measles and rubella elimination in Eswatini by 2024

**DOI:** 10.1371/journal.pgph.0006860

**Published:** 2026-07-16

**Authors:** Xolisile Dlamini, Tholokwakhe Simelane, Susan Kamalizeni, Lonkululeko Khumalo, Angel Dlamini, Kassahun Mitiku, Balcha Masresha

**Affiliations:** 1 Expanded Programme on Immunization, Ministry of Health, Mbabane, Eswatini; 2 National Health Laboratory Service, Mbabane, Eswatini; 3 World Health Organization, country office, Mbabane, Eswatini; 4 Independent consultant, Addis Ababa, Ethiopia; 5 World Health Organization, Regional office for Africa, Brazzaville, Congo; University of Ottawa, CANADA

## Abstract

Eswatini has been implementing measles and rubella elimination strategies, aligning with the WHO African Regional goal. This report reviews the immunization coverage and surveillance data to evaluate the country’s progress towards the elimination goals. We reviewed routine immunisation coverage data, supplemental immunisation activity coverage data, and case based and laboratory surveillance data for measles and rubella. Routine immunization coverage for the first and second dose was 85% and 82% respectively in 2023 according to the WHO UNICEF coverage estimates. Eswatini implemented regular nationwide preventive supplemental immunisation activities, with the most recent one in 2021 attaining 97% administrative coverage. Eswatini has consistently met the performance targets for the principal indicators since 2008. The incidence of both measles and rubella have been maintained at less than 5 cases per million for more than 10 years, with zero incidence of measles documented over multiple years. Eswatini has made immense progress towards measles and rubella elimination. The country will need to address programmatic gaps that may negatively impact population immunity and will need to maintain high quality measles and rubella surveillance, including molecular surveillance in order to attain and maintain the verification of measles and rubella elimination.

## Introduction

The Kingdom of Eswatini spans an area of 17,364 square kilometers and shares borders with South Africa and Mozambique. Its population in 2024 is approximately 1,202,285 people, projected from the 2017 census. The majority of the population (78%) live in rural areas. Eswatini is classified as a lower-middle-income nation by the World Bank. However, the country faces 29% poverty rate, and 24.8% HIV/AIDS prevalence rate among adults. The child mortality rate is 53 deaths per 1,000 live births in 2022. [1–3].

The Eswatini Expanded Programme on Immunization (EPI) offers a comprehensive immunization schedule that includes the following vaccines: BCG, diphtheria, pertussis, tetanus, hepatitis B, polio, measles, rubella, pneumococcal, hemophilus influenzae b (Hib), rotavirus, human papillomavirus (HPV), and COVID-19 vaccines [1]. Eswatini’s EPI program provides the first dose of measles containing vaccine (MCV1) at 9 months of age since 1982, and the second dose of measles containing vaccine at 18 months of age since 1995. Eswatini introduced measles-rubella (MR) vaccine in 2016. The national immunisation policy promotes opening vials of measles-rubella vaccine at every opportunity even if one child presents to a vaccination session. The national program has been using 5-dose measles-rubella vaccine vials starting in 2018, switching from the 10 dose vials used in previous years. [4]

The country started implementing strategies for the accelerated control of measles since 1998, with the conduct of the first measles preventive vaccination campaign, as part of the concerted efforts among southern African countries, which documented high levels of control within a short period. [5] In 2009 – 2011, seven southern African countries, including Eswatini, experienced a resurgence of measles with a total of 144,580 confirmed measles cases reported in the subregion. This large multi-county outbreak was ascribed to immunity gaps resulting from declining routine immunisation coverage, suboptimal SIAs and vaccine hesitancy among certain populations. [6]

The 47 countries in the WHO African Region currently have adopted a goal for the elimination of measles and rubella in at least 80% of the countries by 2030. [7] As of the end of 2023, in the African Region there has been an estimated 79% reduction in annual measles deaths as compared to the estimates for 2000. [8]. Globally, by 2022, the verification of rubella elimination was achieved in 98 countries while measles was verified to have been eliminated in 82 of 194 countries by 2023. [8,9] In the African Region, as of the end of 2024, no country has attained the verification of elimination of measles or rubella. [10]

This report provides an in-depth analysis of Eswatini’s progress toward measles-rubella elimination.

## Methods

We analysed available immunization coverage data for the years 2014 – 2023, and available disease surveillance data from 2008 – 2024.

### Immunisation coverage

The administrative measles vaccination coverage (MCV1 and MCV2 coverage) results are calculated using the administrative method of dividing the total number of doses administered to children in the target age group by the census-estimated number of children in that age group. Every year, the national administrative vaccination coverage data is reported to the WHO and UNICEF using the joint reporting form (JRF). WHO and UNICEF review the submitted data, as well as any available survey results, and generate annual national estimates of coverage for each antigen and for each country. For this analysis, we used the national MCV1 and MCV2 coverage estimates generated by WHO and UNICEF. [11]

We also reviewed the measles /MR vaccination coverage data (administrative and survey data) from the various supplemental immunisation activities implemented in the country until 2024.

### Case based surveillance performance and epidemiological trends

Eswatini has been implementing measles case-based surveillance starting in 2003. The national measles laboratory, part of the National Molecular Reference Laboratory under the Eswatini Health Laboratory Service, is a member of the Regional and global lab networks and is supported by WHO to maintain standard protocols and tools, as well as undergoes regular external quality assessment and accreditation since 2007.

Eswatini employs a comprehensive, elimination-standard case-based surveillance system for monitoring suspected measles and rubella cases. The country utilizes a case definition of “fever and generalized maculopapular rash” for the notification of suspected measles/rubella cases. Specimens from suspected cases are tested for measles and rubella using the ELISA assay method. The confirmation of measles cases is done on the basis of IgM positivity, epidemiological linkage or clinical compatibility. Rubella cases are confirmed based on rubella specific IgM positivity. [12] We analyzed the measles and rubella case-based surveillance and lab databases for the years 2000–2024. We reviewed the epidemiological pattern of confirmed cases of measles and rubella.

Measles surveillance performance is monitored using standard performance indicators. The two principal performance indicators are: non-measles febrile rash illness rate (target of at least 2 per 100,000 population) and the proportion of districts that have investigated at least one suspected case of measles with blood specimen per year (target at least 80% of districts per year). The incidence of confirmed measles is calculated as a rate per million, by dividing the total number of confirmed measles cases (confirmed by laboratory, epidemiological linkage and clinical criteria) by the total population. For the incidence of rubella, the numerator is the number of laboratory-confirmed rubella cases. [12].

## Results

### Routine Immunization

**Fig 1:**
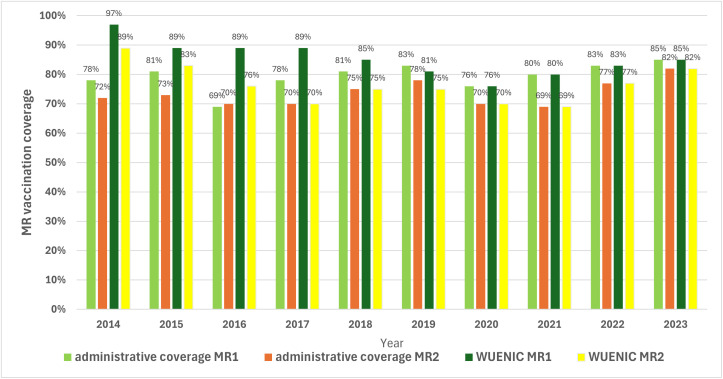
Administrative coverage and WHO-UNICEF coverage estimates (WUENIC) of MCV1 and MCV2 at national level. Eswatini, 2014 – 2023.

The MCV1 coverage was 97% in 2014. Since 2015, the country has seen a decline of MCV1 coverage reaching the lowest level of 76% in 2020. As of 2023, the WHO UNICEF estimates of MCV1 coverage has reached 85%, while the coverage for the measles second dose vaccine (MCV2) is 82%. The administrative MCV1 coverage remained between 69% and 85% during the past 10 years. (Fig 1)

The administrative coverage trends at the provincial level shows a decline in coverage for MCV1 and MCV2 in 2020 and 2021 in all regions, with some recovery in 2023. However by 2023, Manzini and Hhohho regions showed lower coverage levels compared to the other regions (Table 1).

**Table 1:** Administrative measles and rubella vaccination first and second dose coverage by Region. Eswatini. 2019-2023.

Province	2019		2020		2021		2022		2023	
	MR1	MR2	MR1	MR2	MR1	MR2	MR1	MR2	MR1	MR2
**Hhohho**	80%	73%	75%	71%	77%	73%	80%	78%	84%	86%
**Lubombo**	77%	76%	71%	65%	89%	74%	94%	89%	94%	96%
**Manzini**	83%	77%	73%	65%	78%	63%	78%	69%	77%	73%
**Shiselweni**	95%	94%	95%	88%	82%	68%	82%	85%	91%	87%

### Supplementary immunization activities (SIAs)

Eswatini conducted several supplementary immunization activities (SIAs) since 1998 at intervals of 3 – 4 years. The most recent SIA was implemented in 2021 and achieved administrative coverage rate of 97% against rubella and measles. Post-campaign coverage survey was done only in 2013 and in 2016 (Table 2).

**Table 2:** Measles Rubella SIAs administrative coverage by year. Eswatini. 1998 -2021.

Year	Type of SIAs	Target age group	Administrative coverage (%)	Post-campaign coverage survey (%)
1998/99	Measles follow up SIAs	9 – 59 months	94%	–
2002	Measles follow up SIAs	9 – 59 months	86%	–
2006	Measles follow up SIAs	9 – 59 months	91%	–
2009	Measles follow up SIAs	9 – 59 months	96%	–
2010	Measles outbreak response campaign	6 – 59 months	90%	–
2013	Measles follow up SIAs	9 – 59 months	97%	91%
2016	Measles Rubella catch-up SIAs	9 months – 14 years	91%	94%
2021	Measles Rubella follow up SIAs	9 – 59 months	97%	–

### Measles-rubella surveillance

Since 2008, Eswatini has consistently met the annual targets for the two principal measles surveillance performance monitoring indicators (a minimum of 2/100,000 non-measles, non-rubella febrile rash illness rate, and at least 80% of districts achieving the investigation of at least one suspected measles case with a blood specimen per year).

The country experienced a major measles outbreak in 2009–2010 as part of the resurgence in southern Africa, with 28 cases in 2009 and 448 confirmed cases reported in 2010. Between 2011 and 2022, Eswatini did not have any lab confirmed measles cases. Relatively large outbreaks of lab confirmed rubella were reported between 2010 and 2013. In 2023, only three lab confirmed measles cases and one lab confirmed rubella case were reported, while in 2024 the country had 3 laboratory confirmed rubella cases (Table 3).

**Table 3:** Measles and rubella surveillance performance. Eswatini. 2008 – 2024.

Year	Measles suspected cases	Measles confirmed cases	Measles incidence/ million population	Confirmed Rubella Cases	Rubella incidence /million population	Non measles febrile rash illness rate (Target >2/100,000 population)	% Regions Reporting > 1 suspected measles case with blood specimens (Target >80%)
2008	90	1	11.1	0	0	4.6	100
2009	245	28	24	5	4.6	4	100
2010	771	448	407	98	89.1	4.4	82
2011	78	0	0	20	18.1	7.3	100
2012	89	0	0	23	20.7	7.6	100
2013	252	0	0	110	98.4	22.9	100
2014	37	0	0	1	0.9	3.4	100
2015	56	0	0	1	0.9	5.3	100
2016	419	1	0.9	3	2.6	4.7	100
2017	88	0	0	4	3.5	7.6	100
2018	100	0	0	0	3.6	8.7	100
2019	119	0	0	4	0	9.8	100
2020	44	1	0.9	2	3.5	3.7	100
2021	61	1	0.9	1	1.7	5.2	100
2022	61	0	0	0	0.9	5.2	100
2023	122	3	2	1	0	10.3	100
2024	49	0	0	3	2	7.6	100

### Verification of measles and rubella elimination

Eswatini has established the national committee for the verification of measles and rubella elimination, and has updated its progress document in 2024, for presentation to the African Regional Commission for measles and rubella elimination (RVC).

## Discussion

Eswatini continues to demonstrate high level government commitment for preventive maternal and child health services, including for the elimination of vaccine preventable diseases. The government has been fully financing its national immunisation program, including the purchase of vaccines as well as operational funding. The country has made significant progress towards measles and rubella elimination, with sustained high coverage with MCV1. However, coverage has declined since 2017 with further reduction documented during the COVID years, as in many other countries in the Region and beyond. [13–15] The drop-out rate between MCV1 and MCV2 remains quite small at national level since 2018, according to the WHO-UNICEF estimates, and is lower than many other countries in the Region. [10]

According to the 2023 comprehensive EPI review in Eswatini, some of the programmatic challenges include poor health access to the rural populations including limited access for vaccination services; limited human resources and funding for health services; gaps in vaccine and cold chain management leading to vaccine stockouts; increasing vaccine hesitancy fueled by misinformation. [2]

In order to address these gaps, the national immunisation program is implementing integrated outreach services and other community health activities, along with other public health and disease control programs. There are intensive activities implemented to map zero‑dose children and underserved areas on a regular basis to inform scheduled and ad-hoc outreach and mobile vaccination services. In addition, the Ministry of Health is allocating resources to each Public Health Unit to revitalize outreach services to the hard-to-reach areas.

A number of efforts have recently been implemented to address issues related to vaccine hesitancy. These include implementing an updated EPI Social Behaviour Change Strategy; disseminating tailored messages for different immunization audiences, adapted according to the findings from social listening and community dialogues. In addition, the national immunisation program is engaging trusted voices (chiefs, traditional healers, teachers) to counter misinformation. These measures are expected to help the country sustain high vaccination coverage and close immunity gaps, and contribute towards the attainment of measles and rubella elimination.

The country has conducted high quality SIAs in the past, with the most recent campaign implemented in 2021. However, there is a need to plan for the subsequent campaign in order to reach children who missed routine vaccinations. The measles immunity profile shows the percentages of persons immune by MCV1, MCV2, SIAs, and the percentage of persons susceptible to measles by year of birth. [16] By the end of 2025, it is estimated that the number of measles-susceptible children under 5 years of age in Eswatini would be more than the number of children born in the most recent year. This obviously poses a big risk, and requires for the country to implement follow up SIAs targeting under five children. Eswatini is not GAVI eligible, and so is expected to mobilise its own resources to plan and implement these SIAs. The timing and quality of the SIAs will be quite important in terms of closing the immunity gaps and thus averting outbreaks of measles or rubella.

Neighboring South Africa and Mozambique have reported measles outbreaks in 2024, while South Africa experienced a large rubella outbreak with more than 11,000 cases reported in 2024. [17,18] During the huge measles outbreak of 2009 – 2011, a clear propagation pattern was documented from one country to another across southern Africa. [7] Sustaining high population immunity in Eswatini is critical to prevent the importation and local spread of viruses.

With the incidence of measles and rubella at less than 5 per million each for more than a decade, including multiple years of zero incidence, Eswatini is nearing the elimination of both diseases. However, there remain gaps in the quality and depth of epidemiological and molecular investigation and documentation of the few confirmed measles and rubella cases reported in recent years. The verification exercise will help to identify key programmatic elements that need to be addressed to get to the goal. [19] Another country in the subregion, Namibia, has documented a similar and sustained reduction in measles and rubella incidence comparable to Eswatini. [20] In both countries which are in near-elimination settings, it is critical that the sensitivity of surveillance is increased with a view to investigate every measles and rubella case, and all chains of transmission, including with molecular strain characterization.

Our study has some limitations. Incomplete reporting of immunization data from remote or hard-to-reach areas may result in underestimation or inconsistencies in coverage. Inaccuracies in vaccination records may also affect the administrative reported vaccination coverage. In addition, mild cases of measles or rubella may not necessarily be brought to the attention of health workers and not be reported through the surveillance system, especially in remote areas with limited access to health services.

## Conclusion

Eswatini’s achievements towards measles and rubella elimination reflect a comprehensive approach that includes effective vaccination strategies, robust surveillance systems and government commitment. Despite the sustained high population immunity and high-quality surveillance performance, there are some challenges including unreached populations, vaccine hesitancy, delays in implementing time-sensitive preventive vaccination campaigns, and gaps in the detailed investigation of all confirmed measles and rubella cases. Eswatini will need to scale up the measures being taken to address these programmatic challenges as it strives to reach the targets and attain the verification of measles and rubella elimination.
